# Oxygen Extraction and Mortality in Patients Undergoing Chronic Haemodialysis Treatment: A Multicentre Study

**DOI:** 10.3390/jcm12010138

**Published:** 2022-12-24

**Authors:** Silverio Rotondi, Lida Tartaglione, Maria Luisa Muci, Marzia Pasquali, Nicola Panocchia, Filippo Aucella, Antonio Gesuete, Teresa Papalia, Luigi Solmi, Alessio Farcomeni, Sandro Mazzaferro

**Affiliations:** 1 Nephrology and Dialysis Unit, ICOT Hospital, Polo Pontino Sapienza University of Rome, 00185 Rome, Italy; 2 Department of Translation and Precision Medicine, Sapienza University of Rome, 00185 Rome, Italy; 3 Nephrology and Dialysis Unit, Policlinico Umberto I, 00161 Rome, Italy; 4 Department of Nephrology, Fondazione Policlinico Universitario A. Gemelli, IRCCS, 00168 Rome, Italy; 5 Nephrology Unit, Ospedale Casa Sollievo della Sofferenza, 71013 San Giovanni Rotondo, Italy; 6 Nephrology, Dialysis and Transplant Unit, Hospital Annunziata, 87036 Cosenza, Italy; 7 Department of Economics & Finance, University of Rome “Tor Vergata”, 00133 Rome, Italy

**Keywords:** haemodialysis, oxygen extraction, end-stage renal disease, subclinical parenchymal hypoxia, central venous catheter, mortality, central venous oxygen saturation

## Abstract

Patients on haemodialysis (HD) suffer a high mortality rate linked to developing subclinical hypoxic parenchymal stress during HD sessions. The oxygen extraction ratio (OER), an estimate of the oxygen claimed by peripheral tissues, might represent a new prognostic factor in HD patients. This study evaluated whether the intradialytic change in OER (ΔOER) identified patients with higher mortality risks. We enrolled chronic HD patients with permanent central venous catheters with available central venous oxygen saturation (ScvO2) measurements; the arterial oxygen saturation was measured with peripheral oximeters (SpO2). We measured OER before and after HD at enrolment; deaths were recorded during two-years of follow-up. In 101 patients (age: 72.9 ± 13.6 years, HD vintage: 9.6 ± 16.6 years), 44 deaths were recorded during 11.6 ± 7.5 months of follow-up. Patients were divided into two groups according to a 40% ΔOER threshold (ΔOER < 40%, n = 56; ΔOER ≥ 40%, n = 45). The ΔOER ≥ 40% group showed a higher incidence of death (60% vs. 30%; *p* = 0.005). The survival curve (log-rank-test: *p* = 0.0001) and multivariate analysis (*p* = 0.0002) confirmed a ΔOER ≥ 40% as a mortality risk factor. This study showed the intradialytic ΔOER ≥ 40% was a mortality risk factor able to highlight critical hypoxic damage. Using a ΔOER ≥ 40% could be clinically applicable to characterise the most fragile patients.

## 1. Introduction

Patients with end-stage renal disease (ESRD) undergoing chronic haemodialysis (HD) therapy have an increased risk of mortality secondary to the high prevalence of classical cardiovascular risk factors, the presence of risk factors characteristic of chronic kidney disease [1], and nonphysiological HD treatments [2]. HD treatment involves rapid changes in the blood volume and electrolytes, which can result in transient but repeated alterations of organ functions [3]. These alterations are attributable to the need for some parenchyma to increase their metabolic activity during the HD session, with an increase in O_2_ consumption. However, this is associated with a reduced availability of O_2_ linked to the relative hypovolaemia secondary to the rate of ultrafiltration and dialysis-related changes. In particular, alkalinization of the peripheral blood and a reduction of the erythrocyte diphosphoglycerol concentration modify the dissociation curve of haemoglobin, increasing its affinity for O_2_, with a consequent reduction in the O_2_ delivery from haemoglobin to the parenchyma [4,5]. Therefore, regardless of the development of symptoms, an imbalance between O_2_ demand and availability during the HD session results in subclinical parenchymal hypoxia, which causes acute organ function changes, such as myocardial stunning and reduced cerebral perfusion, that are related to long-term organ damage [6,7] and mortality [8]. Accordingly, a parameter able to show and estimate parenchymal hypoxia during HD might be very useful for identifying patients who develop greater intradialytic fatigue, which results in a greater clinical risk. Recently, the oxygen extraction ratio (OER), i.e., the ratio between the arterial oxygen saturation (SaO2) and central venous oxygen saturation (ScvO2), has been proposed as a monitoring system to underline the development of intradialytic subclinical parenchymal hypoxia in HD patients with a central venous catheter since this measurement is only possible in patients with CVC. In any case, the percentage of patients requiring this type of vascular access for long term haemodialysis treatment is increasing, reaching an average prevalence rate of 60% in a recent retrospective study [9]. Two monocentric studies have shown that the OER increases rapidly during HD sessions and that the extent of its intradialytic change (ΔOER) is associated with an increased risk of intradialytic hypotension and mortality [10,11]. These pilot monocentric studies in small populations propose OER as a tool for monitoring the intradialytic stress and identifying an increased clinical risk in patients undergoing HD treatment via a central venous catheter (CVC). At present, large-population clinical studies able to confirm this hypothesis are lacking.

This study aimed to evaluate whether the intradialytic ΔOER could identify patients with a higher mortality risk in a large population of HD patients with CVCs.

## 2. Methods

We conducted a prospective, multicentre, observational study involving patients receiving HD treatment thrice weekly by means of a CVC. The study protocol was approved by the local ethics committee (prot. N°850/18, Sapienza University ethics review board). Informed consent was obtained from all eligible patients. The inclusion criteria were an age ≥ 18 years, undergoing chronic HD treatment for at least 3 months via a permanent jugular CVC, and no evidence of an acute underlying illness nor of any active cancer. The exclusion criteria were less than 3 months of follow-up, presence of an arteriovenous fistula, evidence of a displaced or malfunctioning CVC (checked with chest radiography and CVC recirculation tests), chronic obstructive pulmonary disease, or a peripheral oxygen saturation (SpO2) <90% in the resting condition. In addition, patients with severe refractory anaemia (haemoglobin [Hb] <9 g/dl despite adequate erythropoietin administration and iron supplement therapy), congestive heart failure (New York Heart Association [NYHA] class ≥ II), and severe peripheral vascular ischaemia were excluded. The planned recruitment time was 1 month, and the follow-up time was 2 years. We evaluated OER monthly in the first HD session of the week (after the long interdialytic interval) before and after HD during the 2 years of follow-up. During the follow-up, we recorded deaths and their causes in the study population. OER and ΔOER, the difference between the OER after and before HD, were calculated using the following formulae:

OER=[(SpO2−ScvO2)/SpO2]×100

ΔOER=[(OERTx−OERT0)/OERT0)]×100,

where T0 is the pre-HD OER and Tx is the value obtained at the end of the HD session.

To evaluate the relationship between ΔOER and the mortality risk, we divided the population according to the ΔOER value calculated at the first measurement after inclusion in the study. The threshold ΔOER was 40%, as previously identified [11]. SpO2 levels were monitored using a peripheral pulse oximetry device. Patients wore a finger oximeter during the HD OER sessions, and SpO2 values were recorded at established times. The blood sample to evaluate the pre-HD ScvO2 was drawn from the arterial line of the CVC after discarding 20 mL of blood and before connecting to the extracorporeal circuit. The post-HD ScvO2 was sampled from the arterial line of the dialysis circuit at the end of the dialysis session. Blood gas analysis was immediately performed using dedicated equipment. To avoid pre-analytical artefacts, the handling of the blood for gas analysis was standardised according to the manufacturer’s instructions. Blood for biochemical analysis was sampled at the beginning of the hemodialysis session when OER was measured for the first time. All patients received standard bicarbonate dialysis, according to their individual prescriptions of electrolyte concentrations, dialysate temperature (35.5–36.0 °C), and blood and dialysate flows. The HD OER sessions lasted 4 h, during which the patients fasted. The dialyser membrane was polyarylethersulfone, with a surface tailored to the patient’s body surface. All patients were connected to the extracorporeal circuit without initial haemorrhage.

### 2.1. Ethical Standards

All procedures involving humans in this study were conducted in accordance with common and standard clinical practice, institutional and/or national research committees, and the 1964 Helsinki Declaration and its later amendments or comparable ethical standards.

### 2.2. Statistical Analysis

Data are expressed as mean ± standard deviation (SD) or median ± inter quartile range (IQR). We evaluated the association of OER with time-to-event outcomes from the date of first HD treatment after the enrolment through stratified Kaplan–Meier curves and associated log-rank tests and/or univariate Cox regression models. Multivariate analyses used Cox regression models, where the final set of predictors was selected by means of forward selection based on the Akaike information criterion, subject to an upper bound of three predictors to be included. The upper bound was used to have at most fifteen events per predictor. All tests were two tailed, and (adjusted) *p*-values < 0.05 were considered statistically significant. The final multivariate model actually included only two predictors. All analyses were performed using the open-source software package R version 4.2.2.

## 3. Results

Between 1 January 2019 and 31 January 2019, we evaluated 140 patients with CVCs from 4 Italian HD centres. A total of 101 patients (age, 72.9 ± 13.6 years; HD vintage, 9.6 ± 16.6 years; 61 males and 40 females) were included in the study. Thirty-nine patients were excluded for the presence of an arteriovenous fistula (n = 10), SpO2 < 90% in the resting condition (n = 9), and <3 months of follow-up (n = 20).

**Figure 1:**
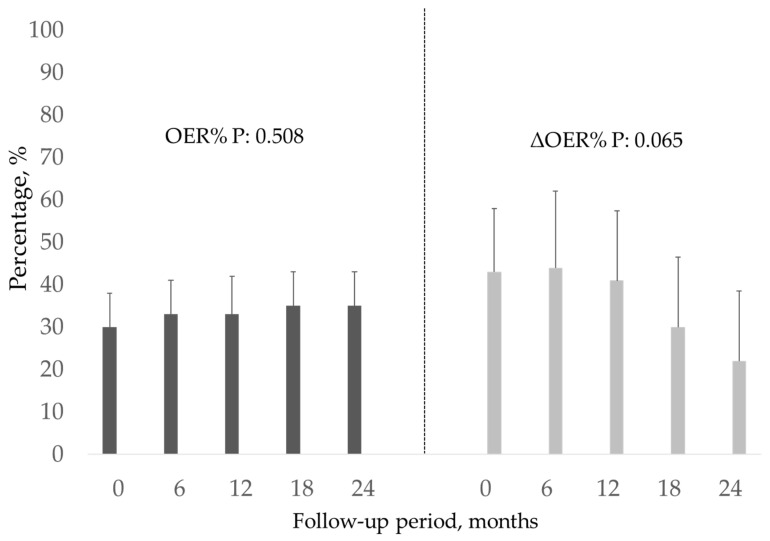
OER pre-HD and ΔOER during the follow-up. OER, oxygen extraction ratio; ∆OER, variation in OER.

**Figure 2:**
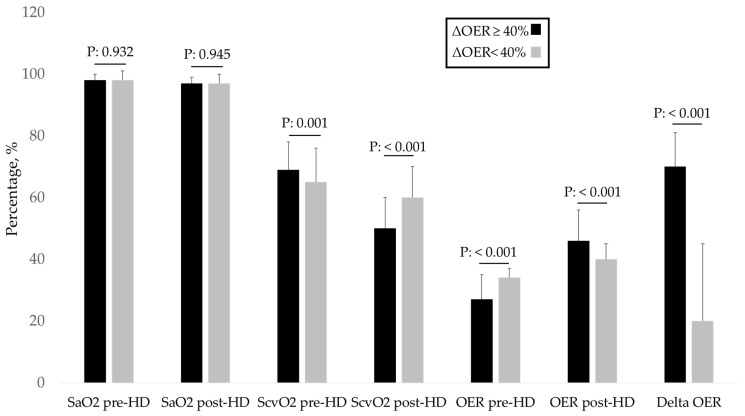
Oxygen parameter in the two groups ΔOER ≥ 40% and ΔOER < 40%. ScvO2, central venous SO2; SaO2, arterial SO2; OER, oxygen extraction ratio; ∆OER, variation in OER.

**Figure 3:**
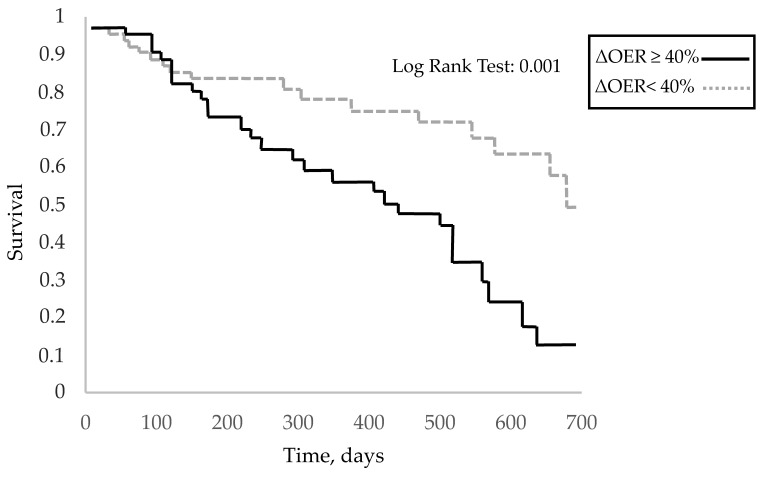
Overall survival, ΔOER ≥ 40% vs. ΔOER < 40%. Time 0 was the date of the first haemodialysis session after enrolment. Kaplan–Meier log rank test, *p* = 0.001. OER, oxygen extraction ratio; ∆OER, variation in OER.

**Figure 4:**
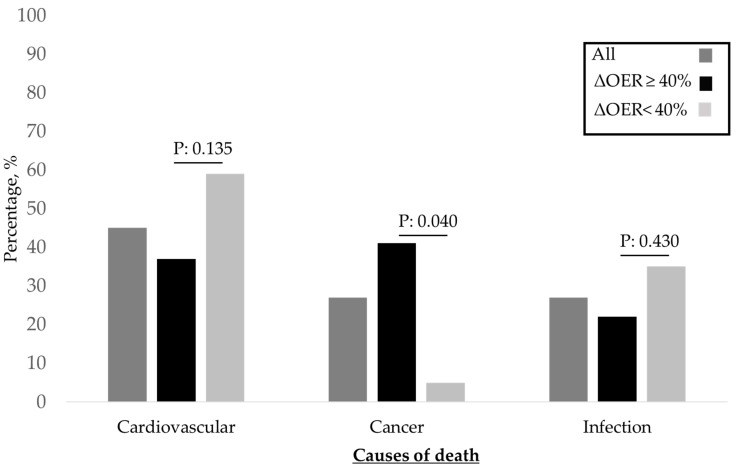
Incidence of causes of death in the entire population and in the ΔOER ≥ 40% and ΔOER < 40% groups. OER, oxygen extraction ratio; ∆OER, variation in OER.

The 101 patients, whose characteristics are shown in Table 1, were followed up for 11.6 ± 7.5 months. Of these, 32% had diabetes, 70% had arterial hypertension, and 44% had previous vascular disease (defined as hypertension, ischaemic heart disease, and peripheral vasculopathy). In this population, at the first measurement after enrolment, the pre-HD OER was 30.8 ± 8.1 (normal OER averages 20–30%) with a mean ΔOER of 42.3 ± 34.8% (Table 1). In the monthly assessment of OER during follow-up, the pre-HD OER and ΔOER values were stable (Figure 1). As required by the protocol, to assess the role of the intradialytic increase in the OER (ΔOER) on mortality, we divided the population into two groups based on baseline measured ΔOER values (threshold of 40%). The characteristics of the two groups are presented in Table 1. The two groups (ΔOER ≥ 40%, n = 45; ΔOER < 40%, n = 56) did not differ in age, dialysis vintage, and prevalence of comorbidities. The evaluated haemodynamic, biochemical, and dialysis efficiency parameters were also not significantly different between the two groups (Table 1). The ΔOER ≥ 40% group had a higher pre-HD ScvO2 (69.3 ± 11.3 vs. 65.4 ± 9.5; *p* = 0.001), lower post-HD ScvO2 (50.5 ± 10.5 vs. 60.3 ± 10.5; *p* < 0.001), lower pre-HD OER (27.1 ± 5.6 vs. 33.8 ± 8.6; *p* < 0.001), and higher post-HD OER (46.1 ± 9.7 vs. 39.8 ± 10.1; *p* < 0.001) (Table 1 and Figure 2). During the follow-up, 27 patients (60%) died in the ΔOER ≥ 40% group, compared with 17 patients (30%) in the ΔOER < 40% group (*p* = 0.005, Table 1). The survival curve confirmed a lower survival in the ΔOER ≥ 40% group (log-rank test: *p* < 0.001, Figure 3). The causes of death had different prevalence rates in the two groups (Figure 4), with a greater prevalence of cancer deaths in the ΔOER ≥ 40% group (*p* = 0.04, Figure 4).

The multivariate analysis showed that a ΔOER of ≥40% (*p* = 0.0002) and heart rate (*p* = 0.001; Table 2) were the best independent predictors of mortality risk in the study population.

The characteristics of the 44 patients who died are shown in Table 3. The 44 non-survivors were compared with survivors and showed lower dialysis vintage (4.5 ± 8.1 vs. 14.7 ± 21.1, *p* = 0.01), higher heart rates before (72.7 ± 9.8 vs. 67.9 ± 11.1, *p* = 0.03) and after HD (75.4 ± 9.2 vs. 68.9 ± 10.2, *p* = 0.003), and lower albuminemia levels (3.2 ± 0.4 vs. 3.5 ± 0.3, *p* = 0.001). Regarding the oxygen parameters evaluated, the non-survival group had a lower post-HD ScvO2 (52.3 ± 11.2 vs. 58.4 ± 10.2, *p* = 0.01) and post-HD OER (45.5 ± 9.8 vs. 39.9 ± 10.4, *p* = 0.01) and a greater dialytic ΔOER (45.3 ± 25.7 vs. 40.0 ± 36.0, *p* = 0.02) than those in the survival group (Table 3).

## 4. Discussion

This prospective multicentre study showed that a ≥40% increase in oxygen extraction during HD, measured using ΔOER%, identified patients with an increased risk of mortality during an average follow-up of 11.6 months.

This study enrolled 101 patients (average age, 72.9 years) with CVCs undergoing chronic HD treatment for 9.6 ± 16.6 years (Table 1) and evaluated the pre- and post-HD OER to obtain the intradialytic ΔOER. OER is obtained from the ratio of the central venous saturation to the arterial oxygen saturation, which measures the O2 parenchymal extraction better than the ScvO2 alone. In fact, the ScvO2 might change even in the case of arterial hypoxia, and in this case, its change does not represent a measure of parenchymal O_2_ extraction, especially in clinical conditions where SaO2 might change, as occurs during HD sessions [12]. For these reasons, OER is a more reliable parameter for monitoring parenchymal oxygen needs and use in HD patients [10]. In our population, the pre-HD OER was 30.8 ± 8.1%, which was within the normal range (20–30%). However, the OER increased during HD sessions, with a post-HD OER of 42.3 ± 13.8% and a ΔOER of 42.3 ± 34.8% (Table 1). This result confirms that during HD sessions, there is an increase in the extraction of parenchymal O_2_, characterised by a stability of the arterial oxygen saturation and a reduction of the ScvO2 (Table 1). This result is consistent with the evidence in the literature showing that hypoxic parenchymal stress develops during HD sessions [13,14]. This hypoxic parenchymal stress requires an increased oxygen extraction, which can be monitored using the OER [8]. Importantly, all OER sessions in the patients were in the absence of symptoms and evident changes in the blood pressure and heart rate (Table 1). It is important to note that OER and ΔOER values were stable over time (Figure 1) in our population, as already evidenced in previous studies [11]. This stability is crucial for rendering OER a clinically useful monitoring parameter. During the follow-up of 11.6 months, we recorded 44 deaths in the population, with an annual mortality of 20%. Of the deaths, 45%, 27%, and 27% were from cardiovascular, infectious, and neoplastic causes, respectively (Figure 4). This result is consistent with what is known in the literature on CVC HD patients, both in terms of the absolute mortality and the incidence of different causes of death [15]. Thus, our study population is representative of the general CVC HD patients in terms of mortality and causes of death. The main purpose of our study was to assess whether a high intradialytic ΔOER (40% threshold) is a risk factor for mortality in HD patients. This hypothesis is derived from the evidence that the hypoxic tissue stress that develops during an HD session causes alterations of organ functions, resulting in chronic organ damage and increased mortality [8]. In agreement with Chan et al., patients at greater clinical risk were identified as those with a greater ScvO2 reduction [16]. Furthermore, a pilot study of 20 patients identified the intradialytic ΔOER as a possible risk factor for mortality [11]. To verify the above hypothesis, we divided our population of 101 patients into two groups based on the first ΔOER value measured after enrolment in the study. The limit of the ΔOER used to identify the two groups was decided by the protocol to be 40% based on the data in the literature [11]. We dichotomised our population into two groups: ΔOER ≥ 40 and ΔOER < 40%. As shown in Table 1, the two groups were clinically similar (same age, dialytic history, comorbidity, and dialytic efficiency), but the group with a ΔOER of ≥40% had a mortality of 60%, compared with 30% in the group with a ΔOER of <40% (*p* = 0.005, Table 1). This was confirmed by the survival curve (Figure 3), which showed that patients with a ΔOER of ≥40% had increased mortality (log-rank test: *p* = 0.001), and multivariate analysis, which highlighted a ΔOER of ≥40% as an independent risk factor in the studied population (Table 2). These results agree with other evidence confirming that patients who need to significantly increase oxygen extraction by the parenchyma during HD have a higher mortality risk, probably secondary to the increased chronic hypoxic parenchymal damage linked to the haemodialytic stress [17]. The change in oxygen extraction in our patients was not caused by a change in SaO2, which remained unchanged (Table 1), but by an increase in the parenchymal extraction, which resulted in a reduction of the ScvO2 and therefore an increase in the OER (Figure 2). An interesting fact that confirms what has already been highlighted in the literature [11] is that the ΔOER ≥ 40% group had a lower pre-HD OER (Table 1, Figure 2). These data may suggest that these patients have a lower adaptation to the uraemic condition. In fact, patients with ESRD have a chronic impairment of the ability to use and distribute O_2_ secondary to a reduction of the capillary bed and mitochondrial dysfunction [8,18,19]. According to various studies, the basal ScvO2 of patients undergoing HD is lower than expected because of the increased need for basal oxygen extraction in this population [17]. The presence of normal O_2_ extraction in a uraemic population can indicate a maladjustment to the condition, resulting in a large increase in the O_2_ extraction during the HD session. Patients who developed lower hypoxic stress (ΔOER < 40% group) during the haemodialytic session had a basally increased OER, a sign of a better adaptation to the uraemic state (Figure 2). Interestingly, the causes of death in the two groups studied (ΔOER ≥ 40% and <40%) had different incidences (Figure 4). In particular, the group with a ΔOER of ≥40% had an increased risk of mortality due to neoplastic causes (Figure 4, *p* = 0.04). These data are in agreement with the experimental evidence showing that intermittent hypoxia, such as that present in our patients, increases the risk of cancer [20,21]. This explains, at least in part, the increased presence of cancer in the group with increased intradialytic parenchymal hypoxia (Figure 4).

Retrospectively assessing the differences between the non-survival (n = 44) and survival (n = 57) populations showed that non-survivors had a shorter dialysis history, lower albuminemia, and a higher average ΔOER and heart rate (Table 3). These data confirm some well-known risk factors in HD patients, such as low albuminemia and a high heart rate [22,23]. In particular, one of the parameters that changes in the case of tissue hypoxaemia is the heart rate, and our data showed that patients at greater risk of mortality had a higher heart rate and higher oxygen extraction during the HD treatment. According to our data, we identified two types of patients: those adapted to the uraemic state and those that were not adapted or maladjusted. The characteristics of this last typology of patients, with an increased risk of mortality, are an intradialytic ΔOER ≥ 40%, a lower pre-HD OER, a higher basal heart rate, and a shorter HD history. A major limitation of the OER measurement technique is that it can be exclusively applied to patients with CVC and without a fistula. However, recent evidence showed that CVC use in HD is nonetheless necessary in 50–60% of patients with poor vascular systems who are also fragile and have higher mortality rates (9). In these patients, the ΔOER could be an easily measurable parameter applicable in clinical practice to highlight the most fragile patients. This would allow us to act on these patients with targeted therapies. Another limitation of the study was that, due to the observational nature of the protocol, it was not possible to verify if the intradialytic ΔOER could be reduced by therapeutic interventions (e.g., modulation of intradialytic ultrafiltration, pre-HD physical activity, pharmacological heart rate modulation). This is a very interesting and important point and future interventional studies are needed to assess whether the ΔOER can be reduced with therapeutic actions.

In conclusion, our data showed that patients who develop greater parenchymal hypoxic damage during HD treatment have an increased mortality risk and that the measurement of the OER and intradialytic ΔOER can highlight the development of this critical hypoxic damage.

## Figures and Tables

**Table 1:** Clinical and biochemical characteristics of the enrolled population and of the two sub-groups (ΔOER ≥ 40% and ΔOER < 40%).

	Enrolled Population (n = 101)	ΔOER ≥ 40% (n = 45)	ΔOER < 40% (n = 56)	*p* Value
Male/female, n (%)	61 (60%)/40 (40%)	28 (62)/17 (38)	33 (58)/23 (42)	0.535 *
Age, years	72.9 ± 13.6	70.6 ± 12.5	74.7 ± 14.3	0.240
Vintage HD, years	9.6 ± 16.7	8.2 ± 15.4	10.7 ± 18.0	0.490
BMI (kg/m^2^)	23.1 ± 2.5	24.9 ± 2.1	25.6 ± 4.1	0.630
Diabetes mellitus, n (%)	32 (32)	15 (33)	17 (30)	0.525 *
HT, n (%)	70 (70)	33 (70)	37 (66)	0.545 *
Vascular comorbidities °, n (%)	44 (44)	20 (44)	24 (43)	0.830 *
IV Erythropoietin therapy, n (%)	94 (93)	42 (92)	52 (93)	0.935 *
IV iron therapy, n (%)	94 (93)	42 (92)	52 (93)	0.935 *
Pre-HD systolic BP, mmHg	131.4 ± 23.1	131.7 ± 27.6	131.1 ± 19.0	0.930
Pre-HD diastolic BP, mmHg	69.6 ± 11.8	71.4 ± 12.2	68.2 ± 11.5	0.275
Post-HD systolic BP, mmHg	133.2 ± 19.7	131.9 ± 18.7	134.3 ± 20.6	0.645
Post-HD diastolic BP, mmHg	71.1 ± 11.3	72.6 ± 10.5	69.9 ± 11.9	0.230
Pre-HD HR, bpm	69.9 ± 10.8	71.6 ± 11.1	68.6 ± 10.5	0.240
Post-HD HR, bpm	71.2 ± 10.4	72.7 ± 10.1	69.9 ± 10.7	0.250
Hb, g/dL	10.6 ± 1.3	10.6 ± 1.5	10.5 ± 1.3	0.730
CRP, mg/dL	1.5 ± 2.0	1.4 ± 2.2	1.8 ± 1.5	0.240
Ferritin, mcg/L	316.9 ± 200.1	320.3 ± 200.5	300.2 ± 150.5	0.155
Albumin, g/dL	3.4 ± 0.4	3.3 ± 0.5	3.4 ± 0.3	0.340
Ca, mg/dL	8.7 ± 0.7	8.7 ± 0.6	8.7 ± 0.7	0.985
P, mg/dL	5.1 ± 1.4	5.3 ± 1.4	5.1 ± 1.4	0.530
PTH, pg/mL	347.9 ± 247.8	360.4 ± 287.6	337.6 ± 212.4	0.740
KT/V	1.3 ± 0.2	1.3 ± 0.2	1.3 ± 0.2	1.000
Total UF, mL	607.1 ± 193.2	632.5 ± 197.4	586.3 ± 188.5	0.435
UF, mL/h/kg	8.3 ± 3.3	8.1 ± 3.2	8.4 ± 3.4	0.650
ScvO2 pre-HD, %	67.1 ± 9.5	69.3 ±11.3	65.4 ± 9.5	0.001
ScvO2 post-HD, %	55.2 ± 11.3	50.5 ± 10.5	60.3 ± 10.5	<0.001
SaO2 pre-HD, %	98.1 ± 3.5	98.5 ± 3.1	98.2 ± 2.1	0.930
SaO2 post HD, %	97.2 ± 3.1	97.0 ± 3.0	97.0 ± 2.0	0.945
OER pre-HD	30.8 ± 8.1	27.1 ± 5.6	33.8 ± 8.6	<0.001
OER post-HD	42.3 ± 13.8	46.1 ± 9.7	39.8 ± 10.1	<0.001
∆OER, %	42.3 ± 34.8	70.4 ± 25.3	19.7 ± 11.6	<0.001
Follow-up, months	11.6 ± 7.5	11.1 ± 6.6	12.1 ± 8.1	0.505
Death, n (%)	44 (44)	27 (60)	17 (30)	0.005 *
Annual mortality rate, %	20	30	15	0.005 *
Causes of death				
Cardiovascular, n (%)	20 (45)	10 (37)	10 (59)	0.135
Cancer, n (%)	12 (27)	11 (41)	1 (5)	0.040
Infection, n (%)	12 (27)	6 (22)	6 (35)	0.430

Data are expressed as mean ± SD. HD, haemodialysis; BMI, body mass index; HT, hypertension; BP, blood pressure; MAP, mean arterial pression; HR, heart rate; Hb, Haemoglobin; CRP, C-reactive protein; Ca, calcium; P, phosphate; PTH, serum parathormone; UF, ultrafiltration rate; ScvO2, central venous SO2; SaO2, arterial SO2; OER, oxygen extraction ratio; ∆OER, variation in OER; IV: intravenous; ° Vascular comorbidities: hypertension, ischaemic heart disease, and peripheral vasculopathy. ΔOER < 40% vs. ΔOER ≥ 40%; * Chi-squared test for qualitative variables. *T*-test for quantitative variables were used to compare measurements between groups.

**Table 2:** Univariate and multivariate survival analysis of the enrolled population (N = 101).

**Univariate Survival Analyses**				
**Variable**	**HR**	**C.I.low**	**C.I.up**	***p***
Age, years	1.01	0.99	1.04	0.360
Vintage HD, years	0.97	0.92	1.02	0.255
OER pre-HD	0.97	0.93	1.01	0.165
OER post-HD	1.02	0.98	1.05	0.295
Delta OER %	1.01	1.00	1.02	0.100
Delta OER % ≥40%	2.76	1.46	5.22	<0.001
Pre-HD systolic BP, mmHg	1.00	0.99	1.01	0.745
Pre-HD diastolic BP, mmHg	1.00	0.97	1.02	0.940
Pre-HD HR, bpm	1.03	1.00	1.06	0.030
Post-HD systolic BP, mmHg	0.99	0.98	1.01	0.475
Post-HD diastolic BP, mmHg	1.00	0.97	1.03	0.930
Post-HD HR, bpm	1.05	1.01	1.08	0.001
Uf h, mL	0.99	0.99	0.99	0.010
Uf mL/min/Kg	0.88	0.80	0.98	0.020
Hb g/dL	0.90	0.71	1.15	0.410
Ca mg/dL	0.86	0.52	1.40	0.540
P mg/dL	0.94	0.74	1.20	0.635
PTH ng/mL	1.00	1.00	1.00	0.490
Diabetes (no-yes)	1.23	0.66	2.31	0.510
CVC (no-Yes)	1.00	0.53	1.89	0.990
CRP	1.00	0.98	1.01	0.990
Albuminemia	1.03	0.99	1.08	0.155
KT/V	0.21	0.04	1.03	0.060
**Multivariate Survival Analyses**				
**Variable**	**HR**	**C.I.low**	**C.I.up**	***p***
Delta OER % ≥40%	2.76	1.46	5.22	<0.001
Post-HD HR, bpm	1.05	1.01	1.08	0.001

∆OER, variation in OER; HD, haemodialysis; BP, blood pressure; HR, heart rate, UF, ultrafiltration rate; Hb, Haemoglobin; CRP, C-reactive protein; ScvO2, central venous, SO2; SaO2, arterial SO2; OER, oxygen extraction ratio; CVC, central venous catheter; UF, ultrafiltration; C.I., confidence interval.

**Table 3:** Characteristics of survivors and non-survivors.

	Survivors (n = 57)	Non-Survivors (n = 44)	*p*-Value
Male/female, n (%)	33 (57)/24 (43)	28 (63)/16 (37)	0.535 *
Age, years	70.4 ± 14.9	75.5 ± 11.8	0.110
Vintage HD, years	14.7 ± 21.1	4.5 ± 8.1	0.010
BMI (kg/m^2^)	25.2 ± 3.1	24.2 ± 2.1	0.835
Diabetes mellitus, n (%)	15 (26)	17 (38)	0.535 *
HT, n (%)	40 (70)	30 (68)	0.520 *
Vascular comorbidities °, n (%)	29 (51)	15 (34)	0.545 *
Pre-HD systolic BP, mmHg	132.3 ± 26.4	130.1 ± 18.1	0.640
Pre-HD diastolic BP, mmHg	70.1 ± 11.6	69.0 ± 12.4	0.661
Post-HD systolic BP, mmHg	135.4 ± 21.4	130.4 ± 17.2	0.210
Post-HD diastolic BP, mmHg	71.0 ± 11.8	71.2 ± 10.7	0.985
Pre-HD HR, bpm	67.9 ± 11.1	72.7 ± 9.8	0.030
Post-HD HR, bpm	68.9 ± 10.2	75.4 ± 9.2	0.003
Hb, g/dL	10.8 ± 1.4	10.2 ± 14.4	0.061
CRP, mg/dL	1.6 ± 1.5	1.6 ± 2.2	0.991
Ferritin, mcg/L	310.2 ± 150.5	310.3 ± 200.5	0.990
Albumin, g/dL	3.5 ± 0.3	3.2 ± 0.4	0.001
Ca, mg/dL	8.8 ± 0.7	8.5 ± 0.6	0.060
P, mg/dL	5.2 ± 1.4	5.1 ± 1.3	0.630
PTH, pg/mL	363.1 ± 294.3	330.5 ± 187.4	0.575
KT/V	1.35 ± 0.2	1.32 ± 0.2	0.230
UF, mL/h	650.1 ± 189.1	551.3 ± 202.0	0.080
UF, mL/h/kg	9.4 ± 2.9	7.2 ± 3.4	0.095
ScvO2 pre-HD, %	68.3 ± 8.5	67.3 ± 10.3	0.835
ScvO2 post-HD, %	58.4 ± 10.2	52.3 ± 11.2	0.010
SaO2 pre-HD, %	98.4 ± 1.8	98.2 ± 2.2	0.990
SaO2 post HD, %	97.5 ± 2.0	97.3 ± 1.8	0.995
OER pre-HD	30.2 ± 8.4	31.6 ± 7.7	0.370
OER post-HD	39.9 ± 10.4	45.5 ± 9.8	0.015
∆OER, %	40.0 ± 36.0	45.3 ± 25.7	0.040
Follow-up, months	12.3 ± 7.5	10.7 ± 7.4	0.275
Causes of death			
Cardiovascular, n (%)		20 (45)	
Cancer, n (%)		12 (27)	
Infection, n (%)		12 (27)	

Data are expressed as mean ± SD. HD, haemodialysis; BMI, body mass index; HT, hypertension; BP, blood pressure; MAP, mean arterial pression; HR, heart rate; Hb, Haemoglobin; CRP, C-reactive protein; Ca, calcium; P, phosphate; PTH, serum parathormone; UF, ultrafiltration rate; ScvO2, central venous SO2; SaO2, arterial SO2; OER, oxygen extraction ratio; ∆OER, variation in OER; ° Vascular comorbidities: hypertension, ischaemic heart disease, and peripheral vasculopathy; * Chi-squared test for qualitative variables. *T*-test for quantitative variables were used to compare measurements between groups.

## Data Availability

Correspondence and requests for materials should be addressed to S.R.

## References

[1] Goodman W.G., London G., Amann K., Block G.A., Giachelli C., Hruska K.A., Ketteler M., Levin A., Massy Z., McCarron D.A. (2004). Vascular calcification in chronic kidney disease. Am. J. Kidney Dis..

[2] Kooman J.P., Katzarski K., van der Sande F.M., Leunissen K.M., Kotanko P. (2018). Hemodialysis: A model for extreme physiology in a vulnerable patient population. Semin. Dial..

[3] Canaud B., Kooman J.P., Selby N.M., Taal M.W., Francis S., Maierhofer A., Kopperschmidt P., Collins A., Kotanko P. (2020). Dialysis-induced cardiovascular and multiorgan morbidity. Kidney Int. Rep..

[4] Soliani F., Davoli V., Franco V., Lindner G., Lusenti T., Parisoli A., Brini M., Borgatti P.P., Mann H., Stiller S. (1990). Intradialytic changes of the oxyhaemoglobin dissociation curve during acetate and bicarbonate haemodialysis. Possible interactions with haemodialysis-associated hypoxaemia. Nephrol. Dial. Transplant..

[5] Sharma S., Brugnara C., Betensky R.A., Waikar S.S. (2015). Reductions in red blood cell 2,3-diphosphoglycerate concentration during continuous renal replacement therapy. Clin. J. Am. Soc. Nephrol..

[6] McIntyre C.W., Burton J., Selby N., Leccisotti L., Korsheed S., Baker C.S., Camici P.G. (2008). Hemodialysis-induced cardiac dysfunction is associated with an acute reduction in global and segmental myocardial blood flow. Clin. J. Am. Soc. Nephrol..

[7] Eldehni M.T., Odudu A., McIntyre C.W. (2015). Randomized clinical trial of dialysate cooling and effects on brain white matter. J. Am. Soc. Nephrol..

[8] Kooman J.P., Stenvinkel P., Shiels P.G., Feelisch M., Canaud B., Kotanko P. (2021). The oxygen cascade in patients treated with hemodialysis and native high-altitude dwellers: Lessons from extreme physiology to benefit patients with end-stage renal disease. Am. J. Physiol. Renal Physiol..

[9] Arhuidese I.J., Orandi B.J., Nejim B., Malas M. (2018). Utilization, patency, and complications associated with vascular access for hemodialysis in the United States. J. Vasc. Surg..

[10] Rotondi S., Tartaglione L., De Martini N., Bagordo D., Caissutti S., Pasquali M., Muci M.L., Mazzaferro S. (2021). Oxygen extraction ratio to identify patients at increased risk of intradialytic hypotension. Sci. Rep..

[11] Rotondi S., Tartaglione L., Muci M.L., Farcomeni A., Pasquali M., Mazzaferro S. (2018). Oxygen extraction ratio (OER) as a measurement of hemodialysis (HD) induced tissue hypoxia: A pilot study. Sci. Rep..

[12] Meyring-Wösten A., Zhang H., Ye X., Fuertinger D., Chan L., Kappel F., Artemyev M., Ginsberg N., Wang Y., Thijssen S. (2016). Intradialytic hypoxemia and clinical outcomes in patients on hemodialysis. Clin. J. Am. Soc. Nephrol..

[13] Harrison L.E., Selby N.M., McIntyre C.W. (2014). Central venous oxygen saturation: A potential new marker for circulatory stress in haemodialysis patients. Nephron Clin. Pract..

[14] Zhang H., Chan L., Meyring-Wösten A., Campos I., Preciado P., Kooman J.P., van der Sande F.M., Fuertinger D., Thijssen S., Kotanko P. (2018). Association between intradialytic central venous oxygen saturation and ultrafiltration volume in chronic hemodialysis patients. Nephrol. Dial. Transplant..

[15] Roca-Tey R., Arcos E., Comas J., Cao H., Tort J. (2016). Starting hemodialysis with catheter and mortality risk: Persistent association in a competing risk analysis. J. Vasc. Access.

[16] Chan L., Zhang H., Meyring-Wösten A., Campos I., Fuertinger D., Thijssen S., Kotanko P. (2017). Intradialytic central venous oxygen saturation is associated with clinical outcomes in hemodialysis patients. Sci. Rep..

[17] Zhang H., Campos I., Chan L., Meyring-Wösten A., Silva L.M.T., Fuentes L.R., Preciado P., Thijssen S., Kooman J.P., van der Sande F.M. (2019). Association of central venous oxygen saturation variability and mortality in hemodialysis patients. Blood Purif..

[18] Amann K., Breitbach M., Ritz E., Mall G. (1998). Myocyte/capillary mismatch in the heart of uremic patients. J. Am. Soc. Nephrol..

[19] Burkhardt D., Bartosova M., Schaefer B., Grabe N., Lahrmann B., Nasser H., Freise C., Schneider A., Lingnau A., Degenhardt P. (2016). Reduced microvascular density in omental biopsies of children with chronic kidney disease. PLoS ONE.

[20] Lee S., Kang H., Kim I., Yeo C., Kim S., Ban W. (2020). Intermittent hypoxia exacerbates tumor progression in a mouse model of lung cancer. Sci. Rep..

[21] Maisonneuve P., Agodoa L., Gellert R., Stewart J.H., Buccianti G., Lowenfels A.B., Wolfe R.A., Jones E., Disney A.P., Briggs D. (1999). Cancer in patients on dialysis for end-stage renal disease: An international collaborative study. Lancet.

[22] Chang Y.-M., Huang Y.-T., Chen I.-L., Yang C.-L., Leu S.-C., Su H.-L., Kao J.-L., Tsai S.-C., Jhen R.-N., Shiao C.-C. (2020). Heart rate variability as an independent predictor for 8-year mortality among chronic hemodialysis patients. Sci. Rep..

[23] Kaysen G.A., Rathore V., Shearer G.C., Depner T.A. (1995). Mechanisms of hypoalbuminemia in hemodialysis patients. Kidney Int..

